# Supplementary material for: Men’s and women’s knowledge of danger signs relevant to postnatal and neonatal care-seeking: A cross sectional study from Bungoma County, Kenya
Source: PLoS One. 2021 May 13;16(5):e0251543. doi: 10.1371/journal.pone.0251543 (PMC8118271; doi:10.1371/journal.pone.0251543)
Supplement: S1 Table — (DOCX) [file pone.0251543.s001.docx]

S1 Table. Women’s and men’s knowledge and practices relating to pregnancy and postnatal care

|  | **WOMEN**  n=348 | | **MEN**  N=82 | |
| --- | --- | --- | --- | --- |
|  | **N** | **%** | **N** | ***** |
| Knowledge of at least one neonatal danger sign  No  Yes | 170  178 | 48.8  51.2 | 41  41 | 50.0  50.0 |
| Knowledge of at least one postpartum danger sign  No  Yes |  |  | 49  33 | 59.8  40.2 |
| Number of antenatal care visits during last pregnancy  <4  ≥4 | 199  128 | 60.9  39.1 |  |  |
| Accompanied female partner to antenatal care during last pregnancy  No  Yes |  |  | 40  42 | 48.8  51.2 |
| Last birth was in a healthcare facility  No  Yes | 72  274 | 20.8  79.2 | 8  73 | 9.9  90.1 |
| Accompanied female partner to delivery during last childbirth  No  Yes |  |  | 27  53 | 33.8  66.2 |
| Place of delivery for last childbirth  Government hospital  Private clinic  Home  Traditional birth attendant  Other | 231  43  48  13  11 | 66.8  12.4  13.9  3.7  3.2 | 64  9  2  6 | 79.0  11.1  2.5  7.4 |
| Birth attendant during last childbirth  Health professional  Traditional birth attendant  Relative or neighbour  Doctor  Midwife or nurse  Traditional birth attendant  Mother | 273  38  25 | 81.3  11.3  7.4 | 13  59  6  2 | 16.2  73.8  7.5  2.5 |
| Newborn experienced problems after last delivery  No  Yes | 212  136 | 60.9  39.1 |  |  |
| Delay in seeking care after noticing newborn was ill  <1 hour  1-6 hours  >6 hours | 59  18  34 | 53.2  16.2  30.6 |  |  |
