# Supplementary material for: Men’s and women’s knowledge of danger signs relevant to postnatal and neonatal care-seeking: A cross sectional study from Bungoma County, Kenya
Source: PLoS One. 2021 May 13;16(5):e0251543. doi: 10.1371/journal.pone.0251543 (PMC8118271; doi:10.1371/journal.pone.0251543)
Supplement: S2 Table — (DOCX) [file pone.0251543.s002.docx]

S2 Table. Factors associated with women’s knowledge of at least one neonatal danger sign

|  | Unadjusted OR (95% CI) | P-value | Adjusted OR (95% CI) | P-value |
| --- | --- | --- | --- | --- |
| Age of woman (years)  <25 (reference)  ≥25 | 4.44 (2.83-6.96) | **<0.001** | 1.67 (0.50-5.62) | 0.408 |
| Woman’s highest level of education completed  Primary school or less (reference)  Secondary school or greater | 4.84 (3.07-7.63) | **<0.001** | 5.65 (1.88-17.04) | **0.002** |
| Man’s age (years)  <30 (reference)  ≥30 | 2.41 (1.35-4.28) | **0.003** | 0.92 (0.26-3.29) | 0.900 |
| Man’s highest education level completed  Primary school or less (reference)  Secondary school or greater | 2.26 (1.29-3.97) | **0.005** | 0.72 (0.22-2.39) | 0.592 |
| Monthly household income (KSh)  <10,000 (reference)  ≥10,000 | 5.92 (3.22-10.87) | **<0.001** | 4.35 (1.74-10.88) | **0.002** |
| Distance to healthcare facility from home  ≤5 kilometres (reference)  >5 kilometres | 0.60 (0.39-0.92) | **0.020** | 0.47 (0.21-1.08) | 0.075 |
| Gravidity  Primigravida (reference)  Multigravida | 3.70 (2.34-5.89) | **<0.001** | 4.66 (1.52-14.36) | **0.007** |
| Age at first pregnancy  <18 (reference)  ≥18 | 8.99 (4.80-16.83) | **<0.001** | 3.24 (1.00-10.52) | **0.050** |
| Shared decision making for health service seeking between mother and male partner  No (reference)  Yes | 3.25 (2.06-5.13) | **<0.001** | 1.43 (0.61-3.36) | 0.417 |
