# Supplementary material for: Men’s and women’s knowledge of danger signs relevant to postnatal and neonatal care-seeking: A cross sectional study from Bungoma County, Kenya
Source: PLoS One. 2021 May 13;16(5):e0251543. doi: 10.1371/journal.pone.0251543 (PMC8118271; doi:10.1371/journal.pone.0251543)
Supplement: S3 Table — (DOCX) [file pone.0251543.s003.docx]

S3 Table. Factors associated with men’s knowledge of at least one neonatal danger sign

|  | Unadjusted OR (95% CI) | P-value | Adjusted OR (95% CI) | P-value |
| --- | --- | --- | --- | --- |
| Age (years)  <30 (reference)  ≥30 | 0.89 (0.34-2.32) | 0.806 | 0.61 (0.13-2.78) | 0.524 |
| Highest level of education completed  Primary school (reference)  Secondary school or greater | 2.26 (0.79-6.42) | 0.128 | 1.21 (0.35-4.16) | 0.767 |
| Female partner’s age (years)  <25 (reference)  ≥25 | 0.89 (0.35-2.28) | 0.810 | 1.05 (0.27-4.03) | 0.948 |
| Female partner’s highest education level completed  Primary school (reference)  Secondary school or greater | 3.07 (1.17-8.03) | **0.022** | 2.52 (0.86-7.44) | 0.093 |
| Monthly household income (KSh)  <10,000 (reference)  ≥10,000 | 3.83 (1.12-13.12) | **0.033** | 4.09 (1.00-16.64) | **0.049** |
