# Supplementary material for: Men’s and women’s knowledge of danger signs relevant to postnatal and neonatal care-seeking: A cross sectional study from Bungoma County, Kenya
Source: PLoS One. 2021 May 13;16(5):e0251543. doi: 10.1371/journal.pone.0251543 (PMC8118271; doi:10.1371/journal.pone.0251543)
Supplement: S4 Table — (DOCX) [file pone.0251543.s004.docx]

S4 Table. Factors associated with women’s healthy care-seeking practices

|  | Associations with attending antenatal care at least 4 times during the most recent pregnancy | | | | Associations with a woman’s most recent birth occurring in a healthcare facility | | | |
| --- | --- | --- | --- | --- | --- | --- | --- | --- |
|  | Unadjusted OR (95% CI) | P-value | Adjusted OR (95% CI) | P-value | Unadjusted OR (95% CI) | P-value | Adjusted OR (95% CI) | P-value |
| Age of woman (years)  <25 (reference)  ≥25 | 2.16 (1.37-3.40) | **0.001** | 1.57 (0.54-4.60) | 0.410 | 0.54 (0.32-0.92) | **0.024** | 0.39 (0.07-2.05) | 0.263 |
| Woman’s highest level of education completed  Primary school or less (reference)  Secondary school or greater | 3.86 (2.37-6.29) | **<0.001** | 2.26 (0.90-5.71) | 0.083 | 3.38 (1.94-5.86) | **<0.001** | 4.24 (1.35-13.30) | **0.013** |
| Man’s age (years)  <30 (reference)  ≥30 | 1.12 (0.63-1.98) | 0.698 | 0.88 (0.29-2.66) | 0.828 | 0.45 (0.20 – 1.00) | **0.049** | 0.93 (0.18-4.92) | 0.936 |
| Man’s highest education level completed  Primary school or less (reference)  Secondary school or greater | 3.13 (1.71-5.73) | **<0.001** | 1.15 (0.40-3.29) | 0.793 | 3.55 (1.84-6.84) | **<0.001** | 0.84 (0.24-2.99) | 0.787 |
| Monthly household income (KSh)  <10,000 (reference)  ≥10,000 | 2.72 (1.50-4.94) | **0.001** | 1.16 (0.50-2.70) | 0.735 | 1.59 (0.78-3.26) | 0.204 | 1.99 (0.65-6.09) | 0.226 |
| Distance to healthcare facility from home  ≤5 kilometres (reference)  >5 kilometres | 0.67 (0.43-1.06) | 0.087 | 0.64 (0.32-1.29) | 0.215 | 0.61 (0.35-1.06) | 0.081 | 0.61 (0.23-1.65) | 0.335 |
| Gravidity  Primigravida (reference)  Multigravida | 1.66 (1.04-2.66) | **0.034** | 0.97 (0.36-2.59) | 0.948 | 0.59 (0.33-1.04) | 0.070 | 1.96 (0.42-9.21) | 0.396 |
| Age at first pregnancy  <18 (reference)  ≥18 | 5.19 (2.62-10.31) | **<0.001** | 2.11 (0.64-7.07) | 0.222 | 1.57 (0.88-2.81) | 0.127 | 2.45 (0.74-8.13) | 0.396 |
| Shared decision making for health service seeking between mother and male partner  No (reference)  Yes | 5.08 (3.13-8.25) | **<0.001** | 2.27 (1.10-4.67) | **0.027** | 1.59 (0.91-2.78) | 0.102 | 1.34 (0.48-3.70) | 0.578 |
| Knowledge of at least one neonatal danger sign  No (reference)  Yes | 4.46 (2.73-7.29) | **<0.001** | ** |  | 3.26 (1.89-5.72) | **<0.001** | ** |  |
| Number of antenatal care visits during last pregnancy  <4 (reference)  ≥4 |  |  |  |  | 5.20 (2.38-11.39) | **<0.001** | ** |  |

**Not included in the multivariate model since it is on the causal pathway
