# Supplementary material for: Men’s and women’s knowledge of danger signs relevant to postnatal and neonatal care-seeking: A cross sectional study from Bungoma County, Kenya
Source: PLoS One. 2021 May 13;16(5):e0251543. doi: 10.1371/journal.pone.0251543 (PMC8118271; doi:10.1371/journal.pone.0251543)
Supplement: S5 Table — (DOCX) [file pone.0251543.s005.docx]

S5 Table. Factors associated with male partners accompanying women to antenatal care during her most recent pregnancy

|  | Unadjusted OR (95% CI) | P-value | Adjusted OR (95% CI) | P-value |
| --- | --- | --- | --- | --- |
| Age (years)  <30 (reference)  ≥30 | 0.45 (0.17-1.22) | 0.117 | 0.42 (0.09-1.82) | 0.243 |
| Highest level of education completed  Primary school (reference)  Secondary school or greater | 0.63 (0.22-1.74) | 0.368 | 0.25 (0.07-0.95) | **0.042** |
| Woman’s age (years)  <25 (reference)  ≥25 | 0.47 (0.18-1.24) | 0.129 | 0.70 (0.18-2.67) | 0.598 |
| Woman’s highest education level completed  Primary school (reference)  Secondary school or greater | 2.08 (0.82-5.28) | 0.122 | 3.45 (1.09-11.28) | **0.036** |
| Monthly household income (KSh)  <10,000 (reference)  ≥10,000 | 1.06 (0.36-3.17) | 0.913 | 1.56 (0.43-5.69) | 0.504 |
| Men’s knowledge of at least one postpartum danger sign  No (reference)  Yes | 1.89 (0.77-4.63) | 0.165 | ** |  |
| Men’s knowledge of at least one neonatal danger sign  No (reference)  Yes | 3.34 (1.35-8.27) | **0.009** | ** |  |

**Not included in the multivariate model since it is on the causal pathway
