# Supplementary material for: Men’s and women’s knowledge of danger signs relevant to postnatal and neonatal care-seeking: A cross sectional study from Bungoma County, Kenya
Source: PLoS One. 2021 May 13;16(5):e0251543. doi: 10.1371/journal.pone.0251543 (PMC8118271; doi:10.1371/journal.pone.0251543)
Supplement: S6 Table — (DOCX) [file pone.0251543.s006.docx]

S6 Table. Factors associated with a woman’s most recent birth occurring in a healthcare facility, as reported by the male partner

|  | Unadjusted OR (95% CI) | P-value | Adjusted OR (95% CI) | P-value |
| --- | --- | --- | --- | --- |
| Age (years)  <30 (reference)  ≥30 | 0.823 (0.15-4.42) | 0.823 | 0.86 (0.07-11.18) | 0.905 |
| Highest level of education completed  Primary school (reference)  Secondary school or greater | 6.44 (1.38-30.06) | **0.018** | 6.26 (0.78-50.06) | 0.084 |
| Woman’s age (years)  <25 (reference)  ≥25 | 0.29 (0.03-2.51) | 0.262 | 0.24 (0.02-3.34) | 0.288 |
| Woman’s highest education level completed  Primary school (reference)  Secondary school or greater | 2.04 (0.47-8.87) | 0.341 | 0.51 (0.06-4.22) | 0.533 |
| Monthly household income (KSh)  <10,000 (reference)  ≥10,000 | 9.39 (1.96-45.08) | **0.005** | 11.99 (1.59-90.40) | **0.016** |
| Men’s knowledge of at least one postpartum danger sign  No (reference)  Yes | 2.09 (0.40-11.08) | 0.385 | ** |  |
| Men’s knowledge of at least one neonatal danger sign  No (reference)  Yes | 1.03 (0.24-4.43) | 0.971 | ** |  |
| Accompanied wife to ANC during last pregnancy  No (reference)  Yes | 1.91 (0.42-8.60) | 0.398 | ** |  |

**Not included in the multivariate model since it is on the causal pathway
