# Supplementary material for: Men’s and women’s knowledge of danger signs relevant to postnatal and neonatal care-seeking: A cross sectional study from Bungoma County, Kenya
Source: PLoS One. 2021 May 13;16(5):e0251543. doi: 10.1371/journal.pone.0251543 (PMC8118271; doi:10.1371/journal.pone.0251543)
Supplement: Attachment — Submitted filename: PONE-D-20-06532_reviewer.pdf [file pone.0251543.s007.pdf]

PLOS ONE

Men’s and women’s knowledge and practices relating to pregnancy and postnatal care: a cross sectional study from Bungoma County, Kenya

--Manuscript Draft--

**Manuscript Number:**	PONE-D-20-06532
**Article Type:**	Research Article
**Full Title:**	Men’s and women’s knowledge and practices relating to pregnancy and postnatal care: a cross sectional study from Bungoma County, Kenya
**Short Title:**	Knowledge and Practices relating to pregnancy in Bungoma County, Kenya
**Corresponding Author:**	Jesse N Gitaka, M.D.<br>Mount Kenya University School of Health Sciences<br>THIKA, KENYA
**Keywords:**	Kenya; maternal health; newborn health; neonatal; danger signs; obstetric emergencies; male involvement; father engagement; male engagement; health knowledge
**Abstract:**	Background: Neonatal and maternal mortality rates remain high in Kenya. Knowledge of neonatal danger signs may reduce the first delay of deciding to seek care. Evidence is emerging on the influential role of fathers in improving maternal and newborn health. This study analysed the factors that determine men’s and women’s knowledge and practices in pregnancy and postnatal care, in order to inform design of future interventions.<br>Methods: A quantitative, cross-sectional study was undertaken in Bungoma County, Kenya. Women who had recently given birth (n=348) and men whose wives had recently given birth (n=82) participated in structured interviews on knowledge and practices relating to pregnancy and postnatal care. Univariate and multivariate logistic regression analyses were performed to investigate associations with key maternal and newborn health outcomes.<br>Results: 51.2% of women and 50.0% of men knew at least one neonatal danger sign, however women knew more individual danger signs than men. In the univariate model, mother’s knowledge of a least one neonatal danger sign was associated with attending antenatal care ≥4 times (OR 4.46, 95%CI 2.73-7.29, p<0.001), facility birth (OR 3.26, 95%CI 1.89-5.72, p<0.001), and male partner accompaniment to antenatal care (OR 3.34, 95%CI 1.35-8.27, p=0.009). Men who completed secondary school or above had lower odds of accompanying their wives to antenatal care (AOR: 0.25, 95%CI: 0.07-0.95, p=0.042). Higher monthly household income (≥10,000KSh (Approx. 100 USD)) was associated with facility delivery (AOR 11.99, 95%CI 1.59-90.40, p=0.009).<br>Conclusion: Knowledge of neonatal danger signs was low, however there was an association between knowledge of danger signs and increased healthcare service use, including male partner involvement in antenatal care. Future interventions should consider the extra costs of facility delivery and the barriers to men participating in antenatal care.
**Order of Authors:**	Emma Roney
	Christopher Morgan
	Daniel Gatungu
	Peter Mwaura
	Humphrey Mwambeo
	Alice Natecho
	Liz Comrie-Thomson
	Jesse Gitaka
**Additional Information:**	
**Question**	**Response**

Powered by Editorial Manager® and ProduXion Manager® from Aries Systems Corporation

**Financial Disclosure**<br><br>Enter a financial disclosure statement that describes the sources of funding for the work included in this submission. Review the submission guidelines for detailed requirements. View published research articles from *PLOS ONE* for specific examples.<br><br>This statement is required for submission and **will appear in the published article** if the submission is accepted. Please make sure it is accurate.<br><br>**Unfunded studies**<br>Enter: *The author(s) received no specific funding for this work.*<br><br>**Funded studies**<br>Enter a statement with the following details:<br>• Initials of the authors who received each award<br>• Grant numbers awarded to each author<br>• The full name of each funder<br>• URL of each funder website<br>• Did the sponsors or funders play any role in the study design, data collection and analysis, decision to publish, or preparation of the manuscript?<br>• **NO** - Include this sentence at the end of your statement: *The funders had no role in study design, data collection and analysis, decision to publish, or preparation of the manuscript.*<br>• **YES** - Specify the role(s) played.<br><br>* typeset	HM AN and JG received funding through the Collaborative Newborn Support Project, which was supported by the UK Department for International Development's "Reducing Maternal and Neonatal Death in Kenya (2013-2018)" programme through the County Innovation Challenge Fund (grant number INN-R1-GA 004) (https://www.gov.uk/government/organisations/department-for-international-development). ER CM and LCT received no specific funding for this work. The funders had no role in study design, data collection and analysis, decision to publish, or preparation of the manuscript.
**Competing Interests**<br><br>Use the instructions below to enter a competing interest statement for this submission. On behalf of all authors, disclose any competing interests that could be perceived to bias this work—acknowledging all financial support and any other relevant financial or non-financial competing interests.<br><br>This statement **will appear in the published article** if the submission is accepted. Please make sure it is	The authors have declared that no competing interests exist.

accurate. View published research articles from *PLOS ONE* for specific examples.<br><br>**NO authors have competing interests**<br><br>Enter: *The authors have declared that no competing interests exist.*<br><br>**Authors with competing interests**<br><br>Enter competing interest details beginning with this statement:<br><br>*I have read the journal's policy and the authors of this manuscript have the following competing interests: [insert competing interests here]*<br><br>* typeset	
**Ethics Statement**<br><br>Enter an ethics statement for this submission. This statement is required if the study involved:<br><br>• Human participants<br>• Human specimens or tissue<br>• Vertebrate animals or cephalopods<br>• Vertebrate embryos or tissues<br>• Field research<br><br>Write "N/A" if the submission does not require an ethics statement.<br><br>General guidance is provided below. Consult the submission guidelines for detailed instructions. **Make sure that all information entered here is included in the Methods section of the manuscript.**	This study was approved by the Mount Kenya University Ethics Review Committee (MKU/ERC/0096). Participants provided written informed consent by signing informed consent forms after the aims and research process were explained to them, prior to undertaking the questionnaire.

Powered by Editorial Manager® and ProduXion Manager® from Aries Systems Corporation

**Format for specific study types**<br><br>**Human Subject Research (involving human participants and/or tissue)**<br>• Give the name of the institutional review board or ethics committee that approved the study<br>• Include the approval number and/or a statement indicating approval of this research<br>• Indicate the form of consent obtained (written/oral) or the reason that consent was not obtained (e.g. the data were analyzed anonymously)<br><br>**Animal Research (involving vertebrate animals, embryos or tissues)**<br>• Provide the name of the Institutional Animal Care and Use Committee (IACUC) or other relevant ethics board that reviewed the study protocol, and indicate whether they approved this research or granted a formal waiver of ethical approval<br>• Include an approval number if one was obtained<br>• If the study involved *non-human primates*, add *additional details* about animal welfare and steps taken to ameliorate suffering<br>• If anesthesia, euthanasia, or any kind of animal sacrifice is part of the study, include briefly which substances and/or methods were applied<br><br>**Field Research**<br><br>Include the following details if this study involves the collection of plant, animal, or other materials from a natural setting:<br>• Field permit number<br>• Name of the institution or relevant body that granted permission	
**Data Availability**<br><br>Authors are required to make all data underlying the findings described fully available, without restriction, and from the time of publication. PLOS allows rare exceptions to address legal and ethical concerns. See the PLOS Data Policy and FAQ for detailed information.	Yes - all data are fully available without restriction

A Data Availability Statement describing where the data can be found is required at submission. Your answers to this question constitute the Data Availability Statement and **will be published in the article**, if accepted.<br><br>**Important:** Stating 'data available on request from the author' is not sufficient. If your data are only available upon request, select 'No' for the first question and explain your exceptional situation in the text box.<br><br>Do the authors confirm that all data underlying the findings described in their manuscript are fully available without restriction?	
**Describe where the data may be found in full sentences. If you are copying our sample text, replace any instances of XXX with the appropriate details.**<br><br>• If the data are **held or will be held in a public repository**, include URLs, accession numbers or DOIs. If this information will only be available after acceptance, indicate this by ticking the box below. For example: *All XXX files are available from the XXX database (accession number(s) XXX, XXX.*).<br>• If the data are all contained **within the manuscript and/or Supporting Information files**, enter the following: *All relevant data are within the manuscript and its Supporting Information files.*<br>• If neither of these applies but you are able to provide **details of access elsewhere**, with or without limitations, please do so. For example:<br><br>*Data cannot be shared publicly because of [XXX]. Data are available from the XXX Institutional Data Access / Ethics Committee (contact via XXX) for researchers who meet the criteria for access to confidential data.*<br><br>*The data underlying the results presented in the study are available from (include the name of the third party*	Data cannot be shared publicly because participants are potentially identifiable based on their individually disaggregated responses to the questionnaire. Data are available from the Mount Kenya University Ethics Review Committee (contact via +254 672 820 000, +254 709 153 000) for researchers who meet the criteria for access to confidential data.

and contact information or URL).<br>• This text is appropriate if the data are owned by a third party and authors do not have permission to share the data.<br><br>* typeset	
Additional data availability information:	

Powered by Editorial Manager® and ProduXion Manager® from Aries Systems Corporation

Manuscript Click here to download Manuscript CNSP manuscript 20200302.docx

Title: Men's and women's knowledge and practices relating to pregnancy and postnatal care: a cross sectional study from Bungoma County, Kenya

Short title: Men's and women's knowledge and practices relating to pregnancy and postnatal care

Authors: Emma Roney[1,2], Christopher Morgan[1,2,3], Daniel Gatungu[4], Peter Mwaura [4], Humphrey Mwambeo [4], Alice Natecho[5], Liz Comrie-Thomson[1,3,6], Jesse Gitaka [4*]

[1] Burnet Institute, Melbourne, Australia

[2] Melbourne School of Population and Global Health, University of Melbourne, Australia

[3] School of Public Health and Preventive Medicine, Monash University, Australia

[4] Directorate of Research and Innovation, Mount Kenya University, Thika, Kenya

[5] Fountain Africa Trust, P.O. Box 1632-50205, Webuye, Bungoma, Kenya

[6] Department of Uro-Gynaecology, Ghent University, Ghent, Belgium

[*] Corresponding author: Jesse Gitaka, email: jgitaka@mku.ac.ke

ABSTRACT

Background: Neonatal and maternal mortality rates remain high in Kenya. Knowledge of neonatal danger signs may reduce the first delay of deciding to seek care. Evidence is emerging on the influential role of fathers in improving maternal and newborn health. This study analysed the factors that determine men's and women's knowledge and practices in pregnancy and postnatal care, in order to inform design of future interventions.

Methods: A quantitative, cross-sectional study was undertaken in Bungoma County, Kenya. Women who had recently given birth (n=348) and men whose wives had recently given birth (n=82) participated in structured interviews on knowledge and practices relating to pregnancy and postnatal care. Univariate and multivariate logistic regression analyses were performed to investigate associations with key maternal and newborn health outcomes.

Results: 51.2% of women and 50.0% of men knew at least one neonatal danger sign, however women knew more individual danger signs than men. In the univariate model, mother's knowledge of a least one neonatal danger sign was associated with attending antenatal care ≥4 times (OR 4.46, 95%CI 2.73-7.29, p<0.001), facility birth (OR 3.26, 95%CI 1.89-5.72, p<0.001), and male partner accompaniment to antenatal care (OR 3.34, 95%CI 1.35-8.27, p=0.009). Men who completed secondary school or above had lower odds of accompanying their wives to antenatal care (AOR: 0.25, 95%CI: 0.07-0.95, p=0.042). Higher monthly household income (≥10,000KSh, approximately US$100) was associated with facility delivery (AOR 11.99, 95%CI 1.59-90.40, p=0.009).

Conclusion: Knowledge of neonatal danger signs was low, however there was an association between knowledge of danger signs and increased healthcare service use, including male partner involvement in antenatal care. Future interventions should consider the extra costs of facility delivery and the barriers to men participating in antenatal and postnatal care.

Keywords: Kenya, maternal, newborn, health knowledge, male involvement

INTRODUCTION

The 2030 Agenda for Sustainable Development emphasises the need to reduce preventable newborn deaths and the maternal mortality ratio.[1] Kenya's neonatal mortality rate (NMR) is 22 deaths per 1,000 live births, and Kenya's maternal mortality ratio (MMR) is 510 deaths per 100,000 live births.[2] This represents a major challenge if the Sustainable Development Goal targets of a NMR of 12 per 1,000 live births or lower, and an MMR of less than 70 per 100,000 live births,[3] are to be met. Moreover, whilst worldwide under-5 child mortality has been declining, NMR has been reducing at a much slower rate, highlighting the need for urgent attention.[4]

Low knowledge of obstetric and neonatal danger signs is widely reported throughout low- and middle-income countries.[5-8] JHPIEGO's birth preparedness and complication readiness framework proposes that increasing knowledge and awareness of these danger signs will improve problem identification, and thus will reduce the first delay of deciding to seek care.[9,10] Furthermore, caregivers' ability to recognise danger signs has been linked with use of antenatal care (ANC) and skilled birth attendance.[7,11,12]

The importance of skilled birth attendance at every childbirth is widely recognised,[11,13] and has been described as the most significant single factor in averting maternal deaths.[14] Most maternal deaths occur at the time of labour, delivery and the immediate postpartum period,[15] with 46% of all maternal deaths and 40% of all stillbirths and neonatal deaths occurring during the period of labour and the day of birth.[16] The importance of ANC is recognised in the World Health Organization's (WHO) revision of global ANC standards; from 2016 recommending

eight ANC contacts throughout pregnancy.[17] However, in low-resource settings often less than half of women attend the pre-2016 recommendation of at least four ANC visits.[8,12,18] ANC is a crucial intervention in improving birth outcomes and reducing neonatal mortality,[19] and is associated with increased likelihood of skilled delivery.[20]

The Kenyan Government's commitment to improving maternal and newborn health outcomes is demonstrated by the 2013 Free Maternity Services Policy,[21,22] whereby maternal health services are delivered at no cost throughout the primary, secondary and tertiary government health sector.[23] Analysis of this policy shows that it has successfully started to increase facility deliveries, however challenges including knowledge and physical accessibility of the facilities still remain: it is clear that additional factors beyond cost affect uptake of facility-based care for pregnancy and childbirth.[24] Our research therefore seeks to understand the additional determinants of ANC use and childbirth occurring in a facility, especially those relating to mothers' and fathers' perspectives.

Male involvement

Since the 1994 International Conference on Population and Development, there has been increasing recognition of the shared rights and responsibilities of women and men in sexual and reproductive health, including the critical role of male partners and fathers in maternal and child health.[3] The WHO has listed male involvement as a key health promotion intervention for maternal and newborn health.[25,26] It is recommended that men are engaged in health services and optimal home practices during pregnancy, childbirth and also after

birth, however the level of evidence for interventions is low; resulting in calls for further research into the impacts of male involvement strategies on health outcomes.[25,27]

Strict definitions of what male involvement entails are not yet broadly agreed,[27-29] however one elementary indicator is whether men participate in antenatal care visits.[28] The WHO recommendations stipulate that male involvement interventions ought to be culturally specific and thus may differ depending on the context.[25,26] An important factor, however, is ensuring that male involvement interventions continue to promote, or at least not detract from, female autonomy and decision-making.[25,26,30] Pregnancy and childbirth are especially complex because these topics are often considered to be women's business (30), but men are often household decision makers in relation to care-seeking.[27,28]

Collaborative Newborn Support Project

The 'Collaborative Newborn Support Project' has been implemented in Bungoma County, Kenya in order to reduce maternal and neonatal mortality. It is an intervention of quasi-experimental design, involving newborn special care units, telehealth, call centre establishment, neonatology training, and community awareness programs.[31] The cross-sectional study reported here forms part of the 'Collaborative Newborn Support Project' and aimed to inform interventions through an assessment of men's and women's knowledge and practices relating to pregnancy and postnatal care.

METHODS

Study design

This is a descriptive cross-sectional survey of women and men involved in the 'Collaborative Newborn Support Project', Kenya.[31]

Study setting

The study was undertaken in Bungoma County, Kenya. Bungoma County is located in Western Kenya, bordering Uganda, and has a population of 1.67 million people.[32] Agriculture, both in the form of subsistence farming and cash crops, is the main form of economic activity, followed by manufacturing and retail services.[31] Data from the 2019 census shows that 11.3% of the population live in urban areas, and 21.6% use mains electricity as their main source of lighting.[33]

Study population

Mothers and fathers from the same geographic area were recruited independently, meaning that the responses to each questionnaire are not linked as mother-father dyads. In total, 8[illegible] men and 348 women participated in the study. Fathers whose female partners had recently delivered were recruited through convenience sampling, from those accompanying their female partners to healthcare clinics, and from men in market centres. Mothers who had recently delivered were recruited at antenatal and postnatal reproductive care units, and in maternal and child health clinics at two health facilities: Bungoma and Webuye hospitals. These facilities were purposively sampled, based on the fact that they are County and Sub-County referral hospitals respectively.

Data collection

Two structured, closed-ended questionnaires were administered, one to the women's sample and the other to the men's sample. These were adapted and abridged from the JHPIEGO birth preparedness and complication readiness tool sample questionnaires.[9] Both questionnaires covered basic socio-demographic factors and asked similar questions about knowledge, attitudes and practices relating to maternal and newborn health. Data were collected by research assistants over a three-month period in 2017. The questionnaires were in English and Kiswahili. Research assistants translated the questions into local dialect whenever necessary. Following collection, the data were transferred to an Access database and archived in Mount Kenya University servers within the Directorate of Research and Innovation.

Data analysis

The data were cleaned and analysed using Stata 13[34] to find summary statistics and to undertake univariate and multivariate logistic regression analyses. Some variables were grouped to dichotomous responses, based on analysis team consensus, to ensure no group was too small for regression analysis. The common approach of interpreting a p-value of less than 0.05 as indicating statistical significance was taken.

Although questions were not completely uniform across the two questionnaires, where possible we used similar variables across both data sets in the analysis to allow the contrast of mothers' and fathers' knowledge and practices. Outcome variables examined in the logistic

regression analysis covered both knowledge and practices. There was insufficient variability in the data to include attitudes in the final analysis.

Univariate associations were tested between outcome variables and hypothesised factors of association, as determined by similar studies and the availability of data from the questionnaires. These were then included in a multivariate model to control for the effects of confounding. Potential confounding factors included in the women's multivariate model were mother's and father's age and education levels, monthly household income, time to healthcare facility, gravidity, age at first pregnancy and shared decision making for health service seeking between mother and male partner. Potential confounding factors included in the men's multivariate model were mother's and father's age and education level, and monthly household income. Outcomes considered to be on the causal pathway between exposure and outcome were included in univariate models, but not in multivariate models.

Ethical considerations

This study was approved by the Mount Kenya University Ethics Review Committee (MKU/ERC/0096). Participants signed informed consent forms after the aims and research process were explained to them, prior to undertaking the questionnaire.

RESULTS

Socio-demographic characteristics

Women's sample

Most women were under 25 years of age (56.9%), with most of their husbands at least 30 years of age (67%). 38.2% were first time mothers and around half had completed secondary school or higher (54.8%). Women reported their husbands as slightly higher educated, with 68.7% having completed secondary school or higher (at least 12 years of formal school). 63.3% lived in households with a monthly income at or above 10,000 KSh (approximately USD100). More than half (59.1%) lived more than 5 kilometres away from the nearest health facility. 38[illegible] of women reported sharing pregnancy and childbirth decision making with their husband, with the rest either making the decision themselves, their husband making the decision without them, or the decision was made by another family member, such as a mother in law (Table 1).

Table 1: Socio-demographic characteristics of the women's and men's samples

Men's sample

Since the women's and men's cohorts were not recruited in the same way they represent comparable, but not matched, populations. Sampled male respondents, along with their wives, tended to be slightly older, more educated and in higher income households than those in the women's sample. 71.9% of men were 30 years of age or older, with most of their wives at least 25 years of age (69.5%). Three quarters of the men had completed secondary school or higher (75.6%), while 65.8% of their wives had completed secondary school or higher. Most households had a monthly income of over 10,000 KSh (80.5%) (Table 1).

Women’s and men’s knowledge and practices relating to pregnancy and postnatal care

Just over half of the women (51.2%) knew at least one neonatal danger sign, with over a third of women reporting that their newborn had experienced problems after their last childbirth (39.1%). Half of those mothers who had experienced a problem sought care within an hour of noticing the newborn was ill (53.2%), however, 30.6% waited for more than six hours before seeking care. Regarding antenatal care, 39.1% of mothers attended at least four times during the last pregnancy. Most women delivered in a healthcare facility (79.2%), be that a government hospital or a private clinic. In the men’s sample, 40.2% of the men knew at least one postpartum danger sign and half (50.0%) knew at least one neonatal danger sign. 51.2% of men accompanied their wife to antenatal care during her most recent pregnancy, either always, most of the time or sometimes. 90.1% of men reported that their wife’s last childbirth was in a healthcare facility, with two thirds accompanying their wife to health facility for delivery (66.2%). Most deliveries occurred in a government hospital (79.0%) and were attended by a nurse or midwife (73.8%) (Table S1 in supplementary materials).

Identification of neonatal danger signs

Figure 1 illustrates that overall the sample of women could identify a larger number of danger signs than the men, although a similar proportion of women and men were not able to identify any neonatal danger signs (48.8% of women, compared to 50.0% of men). Each individual danger sign was identified by a greater percentage of women than men. Some danger signs were identified approximately twice as frequently by women than by men, such

as poor breastfeeding or not able to breastfeed, fast breathing, fever, and difficult to wake, lethargic or unconscious.

Figure 1: Identification of neonatal danger signs: women and men*

Characteristics associated with knowledge of neonatal danger signs

Characteristics associated with women's knowledge

Higher levels of mother's education (AOR: 5.65, 95% CI: 1.88-17.04, p=0.002), higher household income (AOR: 4.35, 95% CI: 1.74-10.88, p=0.002), multigravidity (AOR: 4.66, 95% CI: 1.52-14.36, p=0.007), and older age at first pregnancy (AOR: 3.24, 95% CI: 1.00-10.52, p=0.05) were all significantly associated with mothers' knowledge of at least one neonatal danger sign, after adjusting for confounding (Table S2 in supplementary materials).

Characteristics associated with men's knowledge

Higher household income, at or above KShs 10,000 per month, was significantly associated with men's knowledge of at least one neonatal danger sign (AOR: 4.09, 95%CI: 1.00-16.64, p=0.049) (Table S3 in supplementary materials).

Characteristics associated with health care-seeking practices

Characteristics associated with women's care-seeking

Mothers who shared care-seeking decision making with their husband had increased odds of attending antenatal care at least four times throughout their most recent pregnancy (AOR: 2.27, 95% CI: 1.10-4.67, p=0.027). Mothers who knew at least one neonatal danger sign had

increased odds of attending four or more antenatal visits during their last pregnancy, compared with those who did not know any neonatal danger signs (unadjusted OR: 4.46, 95% CI: 2.73-7.29, p<0.001) (Table S4 in supplementary materials).

Women who completed secondary school or higher had increased odds of birth in a healthcare facility, compared with those who had completed primary school or less, after adjusting for confounding (AOR: 4.24, 95% CI: 1.35-13.30, p=0.013). The univariate analysis for those factors on the causal pathway also demonstrated that there was a significant increase in the odds of the most recent birth occurring in a healthcare facility both for mothers who knew at least one neonatal danger sign (OR: 3.26, 95% CI: 1.89-5.72, p<0.001), and for mothers who attended antenatal care at least four times during their last pregnancy (OR: 5.20, 95% CI: 2.38-11.39, p<0.001) (Table S4 in supplementary materials).

Characteristics associated with men's practices

In the men's sample, both father's and mother's education was associated with men accompanying their wife to antenatal care during her most recent pregnancy. However, these associations were in opposing directions. Men who completed secondary school or higher had an odds ratio of 0.25 (95% CI: 0.07-0.95, p=0.042), meaning they had a 75% decrease in odds of accompanying their wife to antenatal care, compared with counterparts who had completed primary school only. Conversely, men whose wife had completed secondary school or higher had increased odds of accompanying their wife to antenatal care (AOR: 3.45, 95% CI: 1.09-11.28, p=0.036). In univariate analysis, men who knew at least one neonatal danger sign showed increased odds of accompanying their wife to antenatal care during her

most recent pregnancy (AOR: 3.34, 95% CI: 1.35-8.27, p=0.009) (Table S5 in supplementary materials).

Men in households with a monthly income at or above KShs 10,000 had increased odds of their wives delivering in a healthcare facility (AOR: 11.99, 95% CI: 1.59-90.40, p=0.016), compared with those who were in households with monthly incomes below 10,000 KSh, after adjusting for confounding factors (Table S6 in supplementary materials).

DISCUSSION

Overall, knowledge of neonatal danger signs was low, a finding mirrored in similar studies in other low- and middle-income settings, as well as elsewhere in Kenya.[6,7,35,36] Women were better able than men to identify danger signs, especially when asked to name specific danger signs, even though the men's sample tended to be older, more educated, and from higher income households. In general, knowledge of danger signs was associated with wealth and education, as others have also found.[7,35-37] If men are to be actively involved in decision making and healthcare seeking in maternal and newborn health, improving their knowledge of key issues such as danger signs is a necessary starting point. Our regression models show strong associations between women's and men's knowledge of neonatal danger signs and positive healthcare seeking behaviours in pregnancy and postnatal care. Whilst the cross-sectional study design of this research cannot show causation, these associations suggest a correlation between danger signs knowledge and healthcare seeking practices during pregnancy and childbirth.

The results show that knowledge is not adequate in explaining delays in health care seeking among mothers of newborns: 30% of mothers waited over 6 hours after recognising that their newborn was ill before seeking care. Other factors observed to influence health care-seeking in our results echo the findings of others in suggesting that education and wealth are key determining factors in use of antenatal care,[18] and skilled delivery.[12,21,35] In both the men's and women's samples, higher income was associated with both higher knowledge and healthier practices, such as a woman's most recent childbirth occurring in a facility, even after adjusting for confounding factors. Given that Kenya now has free maternity health

services,[22,23] there may be other cost barriers, aside from cost of care itself, at play in the decision to seek care. Future programs may need to consider, for example, the cost of transport, accommodation, and the opportunity cost of missing employment. Our age and parity findings also suggest that health promotion interventions and health communication initiatives should target first time and/or young mothers; this may also present an opportunity for peer-based community learning, such as through group antenatal care,[38] whereby more experienced and older mothers are able to assist in the teaching of danger signs.

This study has highlighted the low proportion of men accompanying their wives to antenatal care, as seen elsewhere.[39] The conflicting findings whereby more educated women had healthier practices, but more educated fathers (in the men's sample) seemed less likely to accompany their wives to antenatal care warrants consideration. It may be that better educated men are more likely to be in formal employment, and programs aiming to increase male involvement may need to consider clinic scheduling that better enables fathers (and mothers) to balance parental responsibilities with paid work requirements, for example by offering ANC sessions on weekends, outside office hours, or close to the workplace. Shifting gender norms and attitudes among men, women, health providers and employers can also be expected to contribute to men's increasing participation in antenatal care, for example by reducing stigma, normalising men's leave from work or flexible working, and providing inclusive antenatal health services that address both parents' reproductive health needs. Further research, particularly qualitative research, on the challenges and barriers men face in attending antenatal care in Bungoma County would prove useful for informing future

program design. For example, other studies in different locations comment on the ridicule men can face upon their arrival at maternal and child health services,[35,40] and also the lack of infrastructure to accommodate men's presence, such as privacy curtains in clinics.[41]

Limitations and strengths

A key limitation of this research is the small sample size of the men's sample, at 82 participants. This meant that confidence intervals were wide and there was often weak evidence of associations since the small sample size reduced the power of the study to detect smaller differences. Women and men were also administered different questionnaires and were sampled in different ways, limiting the potential for direct comparison between the two groups. The regression analysis undertaken used similar outcomes and exposures in order to smooth the differences between the two cohorts.

The primary strength of this research is that it was conceived and led by local Kenyan researchers and thus addressed local priorities. Additionally, this research has combined data from both women and men in one study, which is not widely seen in the existing literature, thus enabling a level of comparison between these two interconnected groups.

CONCLUSION

Overall, knowledge of neonatal danger signs in Bungoma County, Kenya is low among both women and men. Whilst it is suggested that improving knowledge of the neonatal danger signs can reduce the delay in deciding to seek care,[9,10] there still exists a certain disconnect between knowledge translating into practice for some mothers in Bungoma County who did not immediately seek care once realising their newborn was ill.

The key determining factors in men's and women's knowledge and practices relating to pregnancy and postnatal care were education level, income, gravidity and age at first pregnancy. Future interventions, including those in the 'Collaborative Newborn Support Project', must thus consider the extra costs of childbirth occurring in a facility. Furthermore, interventions should address the barriers to men participating in antenatal care, including work commitments and pervasive social and gender norms around pregnancy and child-rearing. Target groups for knowledge-based interventions should focus on primiparous mothers and mothers under the age of 18 at their first pregnancy. These findings have important implications those working in maternal and newborn health in Bungoma County, as well as the overall reduction of maternal and neonatal mortality in Kenya.

Declarations

Availability of data and materials: The datasets used and analysed during the current study are available from the corresponding author on reasonable request.

Competing interests: The authors declare that they have no competing interests.

Funding: The study was funded by the County Innovation Challenge Fund (CICF) grant number CICF-INN-R1-GA 004 to JG. The funders had no role in study design, data collection and analysis, decision to publish, or preparation of the manuscript.

Authors' contributions: JG conceived the study, JG, HM, AN and DG designed the study, ER and CM analysed and interpreted the data and prepared the manuscript. LCT contributed to interpretation of results. JG, DG, PM, HM, AN coordinated field activities and data collection. All authors read and approved the final manuscript.

Acknowledgements: All authors gratefully acknowledge the participation of the men and women who contributed data to this study. MKU acknowledges the support of DfID under County Innovation Challenge Fund for financial support of the project. Burnet Institute acknowledges the contribution to this work of the Victorian Operational Infrastructure Support Program.

References

1. United Nations. Transforming our world: the 2030 agenda for sustainable development (A/RES/70/1), 2015.
2. World Health Organization. Kenya statistics summary. 2019. http://apps.who.int/gho/data/node.country.country-KEN.
3. United Nations Population Fund. Programme of action of the international conference on population development: 20th anniversary edition, 2014.
4. Cooper P. Strategies to reduce perinatal mortality. Lancet Glob Health 2016; 4(1): e6-7.
5. Kibaru EG, Otara AM. Knowledge of neonatal danger signs among mothers attending well baby clinic in Nakuru Central District, Kenya: cross sectional descriptive study. BMC Res Notes 2016; 9(1): 481.
6. Mbalinda SN, Nakimuli A, Kakaire O, Osinde MO, Kakande N, Kaye DK. Does knowledge of danger signs of pregnancy predict birth preparedness? A critique of the evidence from women admitted with pregnancy complications. Health Res Policy Syst 2014; 12: 60.
7. Pembe AB, Urassa DP, Carlstedt A, Lindmark G, Nystrom L, Darj E. Rural Tanzanian women's awareness of danger signs of obstetric complications. BMC Pregnancy Childbirth 2009; 9: 12.
8. Yadeta TA. Antenatal care utilization increase the odds of women knowledge on neonatal danger sign: a community-based study, eastern Ethiopia. BMC Res Notes 2018; 11(1): 845.

9. JHPIEGO. Monitoring birth preparedness and complication readiness: tools and indicators for maternal and newborn health. Maryland, USA: Johns Hopkins University, 2004.

10. Thaddeus S, Maine D. Too far to walk: maternal mortality in context. Soc Sci Med 1994; 38(8): 1091-110.

11. Kifle MM, Kesete HF, Gaim HT, Angosom GS, Araya MB. Health facility or home delivery? Factors influencing the choice of delivery place among mothers living in rural communities of Eritrea. J Health Popul Nutr 2018; 37(1): 22.

12. Tura G, Afework MF, Yalew AW. The effect of birth preparedness and complication readiness on skilled care use: a prospective follow-up study in Southwest Ethiopia. Reprod Health 2014; 11: 60.

13. World Health Organization. WHO recommendations on maternal health: guidelines approved by the WHO Guidelines Review Committee. Geneva: World Health Organization, 2017.

14. World Health Organization. Reduction of maternal mortality. A joint WHO/UNFPA/UNICEF/World Bank statement. Geneva: World Health Organization, 1999.

15. Ronsmans C, Graham WJ, Lancet Maternal Survival Series steering g. Maternal mortality: who, when, where, and why. Lancet 2006; 368(9542): 1189-200.

16. Lawn JE, Blencowe H, Oza S, et al. Every Newborn: progress, priorities, and potential beyond survival. Lancet 2014; 384(9938): 189-205.

17. World Health Organization. WHO recommendations on antenatal care for a positive pregnancy experience. Geneva: World Health Organization, 2016.

18. Banke-Thomas A, Banke-Thomas O, Kivuvani M, Ameh CA. Maternal health services utilisation by Kenyan adolescent mothers: analysis of the Demographic Health Survey 2014. Sex Reprod Healthc 2017; (12): 37-46.

19. Kuhnt J, Vollmer S. Antenatal care services and its implications for vital and health outcomes of children: evidence from 193 surveys in 69 low-income and middle-income countries. BMJ Open 2017; 7(11): e017122.

20. Gabrysch S, Campbell OM. Still too far to walk: literature review of the determinants of delivery service use. BMC Pregnancy Childbirth 2009; 9: 34.

21. Karanja S, Gichuki R, Igunza P, et al. Factors influencing deliveries at health facilities in a rural Maasai Community in Magadi sub-County, Kenya. BMC Pregnancy Childbirth 2018; 18(1): 5.

22. Tama E, Molyneux S, Waweru E, Tsofa B, Chuma J, Barasa E. Examining the implementation of the Free Maternity Services Policy in Kenya: a mixed methods process evaluation. Int J Health Policy Manag 2017; 7(7): 603-13.

23. Pyone T, Smith H, van den Broek N. Implementation of the free maternity services policy and its implications for health system governance in Kenya. BMJ Glob Health 2017; 2(4): e000249.

24. Gitobu CM, Gichangi PB, Mwanda WO. The effect of Kenya's free maternal health care policy on the utilization of health facility delivery services and maternal and neonatal mortality in public health facilities. BMC Pregnancy Childbirth 2018; 18(1): 77.

25. World Health Organization. WHO recommendations on health promotion interventions for maternal and newborn health. Geneva: World Health Organization, 2015.

26. Tokhi M, Comrie-Thomson L, Davis J, Portela A, Chersich M, Luchters S. Involving men to improve maternal and newborn health: A systematic review of the effectiveness of interventions. PLoS One 2018; 13(1): e0191620.

27. Comrie-Thomson L, Tokhi M, Ampt F, et al. Challenging gender inequity through male involvement in maternal and newborn health: critical assessment of an emerging evidence base. Cult Health Sex 2015; 17 Suppl 2: S177-89.

28. August F, Pembe AB, Mpembeni R, Axemo P, Darj E. Community health workers can improve male involvement in maternal health: evidence from rural Tanzania. Glob Health Action 2016; 9: 30064.

29. Onchong'a JM, Were T, Osero JOS. Knowledge, Perception and Level of Male Partner Involvement in Choice of Delivery Site among Couples at Coast Level Five Hospital, Mombasa County, Kenya. Afr J Reprod Health 2016; 20(1): 71-9.

30. Rahman AE, Perkins J, Islam S, et al. Knowledge and involvement of husbands in maternal and newborn health in rural Bangladesh. BMC Pregnancy Childbirth 2018; 18(1): 247.

31. Gitaka J, Natecho A, Mwambeo HM, Gatungu DM, Githanga D, Abuya T. Evaluating quality neonatal care, call Centre service, tele-health and community engagement in reducing newborn morbidity and mortality in Bungoma county, Kenya. BMC Health Serv Res 2018; 18(1): 493.

32. Kenya National Bureau of Statistics. Kenya Population and Housing Census. Volume 1: Population by County and Sub-County. Nairobi: Kenya National Bureau of Statistics, 2019.

33. Kenya National Bureau of Statistics. Kenya Population and Housing Census. Volume 4: Distribution of Population by Socio-Economic Characteristics. Nairobi: Kenya National Bureau of Statistics, 2019.

34. StataCorp. Stata statistical software: release 13. College Station, TX: StataCorp LP, 2013.

35. Nanjala M, Wamalwa D. Determinants of male partner involvement in promoting deliveries by skilled attendants in Busia, Kenya. Glob J Health Sci 2012; 4(2): 60-7.

36. Markos D, Bogale D. Birth preparedness and complication readiness among women of child bearing age group in Goba woreda, Oromia region, Ethiopia. BMC Pregnancy Childbirth 2014; 14: 282.

37. Kabakyenga JK, Ostergren PO, Turyakira E, Pettersson KO. Knowledge of obstetric danger signs and birth preparedness practices among women in rural Uganda. Reprod Health 2011; 8: 33.

38. Sharma J, O'Connor M, Rima Jolivet R. Group antenatal care models in low- and middle-income countries: a systematic evidence synthesis. Reprod Health 2018; 15(1): 38.

39. Tweheyo R, Konde-Lule J, Tumwesigye NM, Sekandi JN. Male partner attendance of skilled antenatal care in peri-urban Gulu district, Northern Uganda. BMC Pregnancy Childbirth 2010; 10: 53.

40. Comrie-Thomson L, Mavhu W, Makungu C, et al. Male involvement interventions and improved couples' emotional relationships in Tanzania and Zimbabwe: 'When we are walking together, I feel happy'. Cult Health Sex 2019: 1-18.

41. Holmes W, Wambo G, Gabong R, et al. 'Because it is a joyful thing to carry a baby': involving men in reproductive, maternal and newborn health in East New Britain, Papua New Guinea. Papua New Guinea Medical Journal 2012; 55.

Click here to download Figure CNSP table 1 20200302.docx

Table 1: Socio-demographic characteristics of the women's and men's samples

	**WOMEN** n=348		**MEN** n=82	
	**N**	**%**	**N**	**%**
Mother's age (years)				
<25	198	56.9	25	30.5
≥25	150	43.1	57	69.5
Husband's age (years)				
<30	74	33.0	23	28.1
≥30	150	67.0	59	71.9
Mother's highest level of education completed				
Primary school or less	157	45.2	28	34.2
Secondary school or greater	191	54.8	54	65.8
Husband's highest education level completed				
Primary school or less	74	31.3	20	24.4
Secondary school or greater	163	68.7	62	75.6
Monthly household income (KSh)				
<10,000	79	36.7	16	19.5
≥10,000	136	63.3	66	80.5
Distance to healthcare facility from home				
≤5 kilometres	141	40.9		
>5 kilometres	204	59.1		
Gravidity				
Primigravida	129	38.8		
Multigravida	209	61.2		
Age at first pregnancy				
<18	85	25.0		
≥18	255	75.0		
Shared decision making for health service seeking between mother and husband				
No	211	61.2		
Yes	134	38.8		

Figure 1 Click here to download Figure CNSP figure 1 20200302.docx

Figure 1: Identification of neonatal danger signs: women and men*

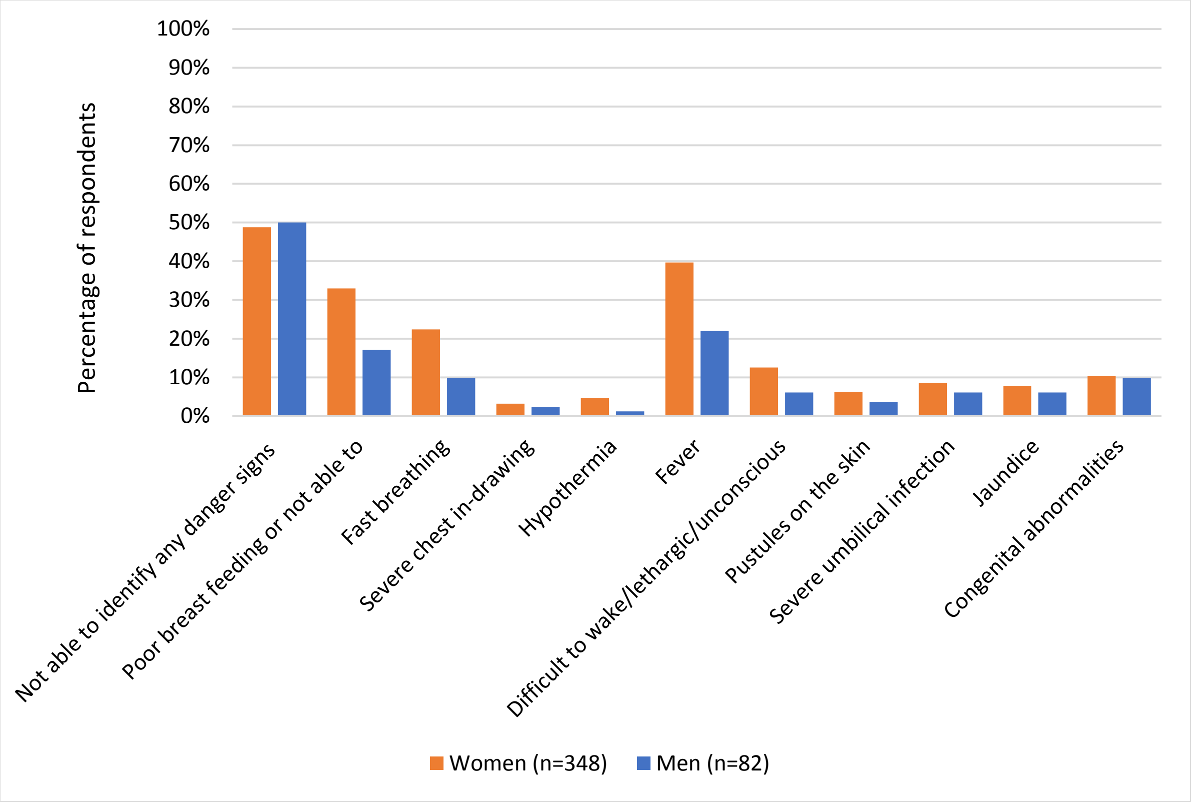

* Note that women's and men's cohorts are comparable, but not matched.

Supplementary tables S1-S6

Click here to access/download
Supporting Information
CNSP supplementary tables 20200302.docx
