# Supplementary material for: Men’s and women’s knowledge of danger signs relevant to postnatal and neonatal care-seeking: A cross sectional study from Bungoma County, Kenya
Source: PLoS One. 2021 May 13;16(5):e0251543. doi: 10.1371/journal.pone.0251543 (PMC8118271; doi:10.1371/journal.pone.0251543)
Supplement: Attachment — Submitted filename: PONE-D-20-06532_reviewer.doc [file pone.0251543.s008.doc]

**PONE-D-06532_reviewer**

**Title:** Men’s and women’s knowledge and practices relating to pregnancy and postnatal care: a cross sectional study from Bungoma County, Kenya

**Short title:** Men’s and women’s knowledge and practices relating to pregnancy and postnatal care

**Comment:** The report is centred on knowledge of newborn danger sign. This should be reflected in the title ie replace “relating to pregnancy and postnatal care”. There are no results on postnatal care

**ABSTRACT:** The authors studied knowledge rather than costs of care. Further studied should address knowledge

**INTRODUCTION**

**Lines 87-89 & 116-117**

“Our research therefore seeks to understand the **additional determinants of ANC use and childbirth** occurring in a facility, especially those relating to mothers’ and fathers’ perspectives.

The cross-sectional study reported here forms part of the ‘Collaborative Newborn Support Project’ and aimed to inform interventions **through an** **assessment of men’s and women’s knowledge and practices** relating to pregnancy and postnatal care.”

**Comment:** It’s difficult to correlate ‘**additional determinants of ANC use and childbirth’ ‘through an** **assessment of men’s and women’s knowledge and practices’**

Indeed the current study there is little information on actual *determinant of use* but the authors mostly report on *determinants of knowledge*

Also give the readers a brief on the broader ‘Collaborative Newborn Support Project’ how long has been going and at stage was the current done

**METHODS**

Study setting does not include the hospitals (for women & men)and the type of markets (for men) where the participants were recruited from.

What was their definition of *1. “recently delivered” 2. “delay in seeking health care for the baby?*

**RESULTS**

**Socio-demographic characteristics &**

**Table 1**

- To avoid confusion in the column for each characteristic, use men or women instead of husband’s or mother’s.
- Give median ages for the women & their partners in both columns.

Do analyses for all the observations to show if there was any significant statistical differences

**Men’s sample:**  In the narrative give the proportion of men who had accompanied their partners in the sample

**Supplementary materials**

**Table S1**

Knowledge of at least one postpartum danger sign – which one?

The authors need to explain why for women data was not reported on the following:

1. Knowledge of at least one postpartum danger sign
2. Accompanied by partner to ANC
3. Accompanied by partner to delivery

**DISCUSSION**

Kenya has a “Mother Child Health Handbook” which is given for each pregnancy and is in taken home by the mother. This handbook has nearly all the information these authors were looking for in the current study, but it is not mentioned at all in the discussion of this paper
