# Supplementary material for: Men’s and women’s knowledge of danger signs relevant to postnatal and neonatal care-seeking: A cross sectional study from Bungoma County, Kenya
Source: PLoS One. 2021 May 13;16(5):e0251543. doi: 10.1371/journal.pone.0251543 (PMC8118271; doi:10.1371/journal.pone.0251543)
Supplement: Attachment — Submitted filename: Response to reviewers.docx [file pone.0251543.s009.docx]

**Response to reviewers**

**2^nd^ Oct, 2020**

Dear Editors,

We are very grateful to the reviewers for their comments. We have comprehensively addressed these and strongly believe that they have enriched the revised manuscript. Kindly see our responses in blue after each comment.

Thank you and best regards,

Jesse Gitaka on behalf or authors.

Journal requirements:

When submitting your revision, we need you to address these additional requirements.

1. Please ensure that your manuscript meets PLOS ONE's style requirements, including those for file naming.

Done

 2. In your Methods section, please provide additional information about the participant recruitment method and the demographic details of your participants for both the womens and mens samples. Please ensure you have provided sufficient details to replicate the analyses such as: a) the recruitment date range (month and year), b) a description of any inclusion/exclusion criteria that were applied to participant recruitment, c) a table of relevant demographic details, d) a statement as to whether your sample can be considered representative of a larger population, e) a description of how participants were recruited, and f) descriptions of where participants were recruited and where the research took place."

Done. See the methods and results sections as well as the supplementary tables.

3. Please provide a sample size and power calculation in the Methods, or discuss the reasons for not performing one before study initiation."

Women and men from the same geographic area were recruited independently, meaning that the responses to each questionnaire are not linked as mother-father dyads. In total, 82 men and 348 women participated in the study. Men whose female partners had recently delivered were recruited through convenience sampling, from those accompanying their female partners to healthcare clinics, and from men in market centres. Women who had recently delivered were recruited at antenatal and postnatal reproductive care units, and in maternal and child health clinics at two health facilities of Webuye level 5 hospital and Bungoma Referral hospital

4. Please include additional information regarding the survey or questionnaire used in the study and ensure that you have provided sufficient details that others could replicate the analyses. For instance, if you developed a questionnaire as part of this study and it is not under a copyright more restrictive than CC-BY, please include a copy, in both the original language and English, as Supporting Information.

 Copies provided

5. We note that you have indicated that data from this study are available upon request. PLOS only allows data to be available upon request if there are legal or ethical restrictions on sharing data publicly. For information on unacceptable data access restrictions, please see <http://journals.plos.org/plosone/s/data-availability#loc-unacceptable-data-access-restrictions>.

In your revised cover letter, please address the following prompts:

a) If there are ethical or legal restrictions on sharing a de-identified data set, please explain them in detail (e.g., data contain potentially identifying or sensitive patient information) and who has imposed them (e.g., an ethics committee). Please also provide contact information for a data access committee, ethics committee, or other institutional body to which data requests may be sent.

We have shared de-identified data at https://doi.org/10.6084/m9.figshare.13048718. Contact information for Mount Kenya University Ethics Review Committee is research@mku.ac.ke.

b) If there are no restrictions, please upload the minimal anonymized data set necessary to replicate your study findings as either Supporting Information files or to a stable, public repository and provide us with the relevant URLs, DOIs, or accession numbers. Please see <http://www.bmj.com/content/340/bmj.c181.long> for guidelines on how to de-identify and prepare clinical data for publication. For a list of acceptable repositories, please see <http://journals.plos.org/plosone/s/data-availability#loc-recommended-repositories>.

We will update your Data Availability statement on your behalf to reflect the information you provide.

 We have uploaded the 2 datasets at https://doi.org/10.6084/m9.figshare.13048718.

6. Please include your tables as part of your main manuscript and remove the individual files. Please note that supplementary tables (should remain/ be uploaded) as separate "supporting information" files

 Done

7. Please include captions for your Supporting Information files at the end of your manuscript, and update any in-text citations to match accordingly. Please see our Supporting Information guidelines for more information: http://journals.plos.org/plosone/s/supporting-information.

 Done

Editor Comments:

Thank you for your revisions. However, there are still many comments from reviewers to address, but we are confident they will make the article stronger.

In addition to those from the reviewer:
-There is inconsistency is the statement of the objective. The title states knowledge, but the paper suggests knowledge, attitudes and practices, and also at times says "determinants of ANC use and facility based childbirth. Please clarify.

The new title captures the attitudes as well.

-Further, the link between knowledge and male involvement isn't very clear.

Indeed this forms part of our research question; what is the best way to address male involvement? What are the knowledge gaps for male involvement?

-The data presented are not very in-depth and it is not clear what this adds to previous literature on this topic, even from Kenya, since there are existing studies. Some of the more nuanced analysis may be in supplemental tables and the authors may want to consider including those info in the main paper.

We have included more tables to deepen the analyses. Our study is unique in that we address contextual issues in a high burden setting.

-There is some inconsistency in language about the questionnaire - in some places it is referred to as an interview.

Corrected.

-I wouldn't say that fathers being influential in maternal and child health is new.

Indeed, this is not new. However we aimed at understanding contextual issues around male involvement in knowledge and practice. In the study context, men have traditionally left child care to women. This perception has been so pervasive that men have been left out in the design of maternal and child health programs and policies. This study hoped to establish a scientific basis for either supporting or refuting these perceptions.

-"recently" delivered needs to be defined (was it deliveries within a year?); also, how were recently delivered women recruited from ANC??

Recently delivered means deliveries within a year. Yes, these were recruited from the ANC.

-the association between knowledge of danger signs and ANC is presented as if knowledge is a factor related to ANC, when it is likely that the association is the other way (those who attend ANC or deliver in a facility learn the danger signs)

This is well understood. However, in our setting, the extended family is also an important source of knowledge on danger signs.

-low knowledge of danger signs is not only in low income countries, but also among those who are disadvantaged/uneducated in high income settings

This is true, but also outside of the scope of this paper, which focuses on Kenya.

-if the authors refer to the "first delay" then the 3 delays model has to be explained

We have taken out the word “first” so as to not complicate things with long explanations of a well-known concept.

-how many danger signs were included in the questionnaire? what were they?

Vaginal bleeding

Neo-natal sepsis

Jaundice

Convulsions

Asphyxia

High fever

Congenital problems

Difficulty breathing

Severe weakness

Accelerated/reduced foetal movement

Water breaks without labour

Bleeding umbilical cord

Poor breastfeeding

Other- open ended.

-For the languages, it if was expected to need other languages besides English and Kiswahili, why not translate ahead of time? it is atypical to use ad hoc translation, as the terminology is then not consistent.

Most of the participants understand Kiswahili, and hence we estimated that there was minimal need to translate to Kibukusu or kisabaoti.

-there is redundant demographic info in the descriptions of the male and female samples

Corrected.

-there should not be new findings presented in the discussion. The results around how long it took to seek care once identifying an illness should be in the results.

This information is presented in both the results table S1 and the body of the text (currently line 289)

-the limitations allude to confidence intervals but these are not presented.

These are in the supplementary tables.

-why were the women's and men's cohorts not recruited the same way? Needs to be explained in the limitations.

Done.

[Note: HTML markup is below. Please do not edit.]

Reviewers' comments:

Reviewer's Responses to Questions

**Comments to the Author**

Reviewer #1: Thank you for the opportunity to review this manuscript, which tackles the important problem of parents knowledge about neonatal danger signs using a cross sectional study from Bungoma County, Kenya.

Is the title reflective of the main message: Men’s and women’s knowledge and practices relating to pregnancy and postnatal care—isn’t it really focused on neonatal danger signs?

Well noted and corrected.

This is a strikingly well written manuscript which has been meticulously copy edited and is easy to read and follow. It makes clear points.

I have a few comments which I will hope to strengthen the manuscript further:
1. In the abstract, why are unadjusted models of mothers’ knowledge presented instead of adjusted ones like for the men?
2. Similarly, in the body of the manuscript in the results section please focus only on the adjusted odds ratio’s and remove the unadjusted ones. Flipping back-and-forth between adjusted and unadjusted ones is confusing to the reader.

We reported on unadjusted odds ratios when the variable was considered to be on the causal pathway, and therefore not included in the multivariate model. This was explained in the methods and is noted in the relevant tables. If we take out the unadjusted ORs, we will lose a big chunk of the data/paper content.

3. Why do the conclusions in the abstract only refer to the mothers’ knowledge?

We have states that “knowledge of neonatal danger signs was low”, which was true for both mothers and men.

4. Please clarify in the introduction when ANC is used whether you mean all of antenatal care or antenatal and intrapartum and postpartum care e.g. particularly in lines 81 - 89, but also elsewhere.

We have clarified that this is during pregnancy.

5. Another key limitation is that we do not know the response rate

The response rate was 88%.

6. In the limitations or strengths could you please mention the degree to which your sample is reflective of the general population in terms of education, age, etc.

Done.

7. For your tables, rather than putting the 1.00 in each of the cells indicating the reference, would you consider decluttering the table by putting (Reference) in the first column only? eg Less than 25 years (Reference)

Done.

8. Similarly, since you were 95% confidence intervals indicate significance you could delete the P values and just bold the odds ratio’s and 95% confidence intervals instead which would decrease clutter within the table and make it easier for your readers to see the main points

We wish to maintain as is, as these complement each other and is the practice generally.

9. Throughout the tables, figures, and manuscript rather than using “father“ please replace with “men” as you do in figure 1 for example. Why? We know from genetics research that 10% of male partners are not actually the father of the child

Done.

10. Please comment on missing data for each question.

We had very few missing data which were treated automatically under regression analyses in stata.

11. Please clarify how missing data was handled e.g. ?complete case analysis

Done.

Minor points:
Please do not start sentences with numbers e.g. line 190

Well noted and corrected.

When you print off your figures in black-and-white, are the two colours easily distinguishable?

Yes.

Reviewer #2: I have made separate comments that need to be addressed. These are scattered through the document so can not be displayed here. The attachment will show the areas needing review by the authors. It is a worthwhile papers that shows where health workers need to focuss on

Reviewer #3: The methodology used should be justified. For example, why is it that male partners of female participants were not enrolled, instead men we conveniently enrolled from the market place. What was the basis of sample size calculation? Why the difference in sample size? What is the role of the collaborative intervention in this study? The clarity of the methodology will help in the clarity of the results, discussion and conclusions. More comments are in the document.

 Done. See methods section.

 Reviewer 2 extended comments:

**Comment:** The report is centred on knowledge of newborn danger sign. This should be reflected in the title ie replace “relating to pregnancy and postnatal care”. There are no results on postnatal care

Well noted and done.

**ABSTRACT:** The authors studied knowledge rather than costs of care. Further studied should address knowledge

Agreed and corrected. Thank you.

**INTRODUCTION**

**Lines 87-89 & 116-117**

“Our research therefore seeks to understand the **additional determinants of ANC use and childbirth** occurring in a facility, especially those relating to mothers’ and fathers’ perspectives.

The cross-sectional study reported here forms part of the ‘Collaborative Newborn Support Project’ and aimed to inform interventions **through an** **assessment of men’s and women’s knowledge and practices** relating to pregnancy and postnatal care.”

**Comment:** It’s difficult to correlate ‘**additional determinants of ANC use and childbirth’ ‘through an** **assessment of men’s and women’s knowledge and practices’**

Indeed the current study there is little information on actual *determinant of use* but the authors mostly report on *determinants of knowledge.*

ANC use is a practice, so we are not being contradictory here. We look at determinants of knowledge and then if knowledge is a determinant of use/practice.

Also give the readers a brief on the broader ‘Collaborative Newborn Support Project’ how long has been going and at stage was the current done.

The Collaborative Newborn Support Project ran from October 2015 to April 2019 and the current study was undertaken July to December 2017.

**METHODS**

Study setting does not include the hospitals (for women & men) and the type of markets (for men) where the participants were recruited from.

What was their definition of *1. “recently delivered” 2. “delay in seeking health care for the baby?*

1. Recently delivered- means had a delivery in the last 1 year.
2. Delay in seeking health care for the baby- Time taken after recognition for need to seek care. We classified this in 3 time bands <1 hour, 1-6 hours and >6 hours.

**RESULTS**

**Socio-demographic characteristics &**

**Table 1**

- To avoid confusion in the column for each characteristic, use men or women instead of husband’s or mother’s.

Done

- Give median ages for the women & their partners in both columns.

Done

Do analyses for all the observations to show if there was any significant statistical differences

We intentionally did not do this because the data are actually not the same.

**Men’s sample:**  In the narrative give the proportion of men who had accompanied their partners in the sample

This is already included under the heading ‘women’s and men’s knowledge and practices relating to pregnancy and postnatal care’ (currently line 292-293)

**Supplementary materials**

**Table S1**

Knowledge of at least one postpartum danger sign – which one?

‘’At least one danger sign” refers to respondents listing one or more of the danger signs in their response and does not limit them to a particular one.

The authors need to explain why for women data was not reported on the following:

1. Knowledge of at least one postpartum danger sign
2. Accompanied by partner to ANC
3. Accompanied by partner to delivery

These weren’t questions in the women’s questionnaire. We do say in the methods that the questionnaires were similar. We have added in slightly more explanation to hopefully make this clearer.

**DISCUSSION**

Kenya has a “Mother Child Health Handbook” which is given for each pregnancy and is in taken home by the mother. This handbook has nearly all the information these authors were looking for in the current study, but it is not mentioned at all in the discussion of this paper

 The Mother Child Handbook captures most of the information we sought, however, in our study we aimed at studying the knowledge, attitudes and practices in our setting in Bungoma County where maternal and newborn indicators are among the worst in Kenya. In revision, we have highlighted the need to enhance utility of the Mother Child Handbook as a source of information on danger signs for families.
