# Supplementary material for: Men’s and women’s knowledge of danger signs relevant to postnatal and neonatal care-seeking: A cross sectional study from Bungoma County, Kenya
Source: PLoS One. 2021 May 13;16(5):e0251543. doi: 10.1371/journal.pone.0251543 (PMC8118271; doi:10.1371/journal.pone.0251543)
Supplement: Attachment — Submitted filename: Response to reviewers, second letter v2.docx [file pone.0251543.s011.docx]

**Second response to reviewers**

***PONE-D-20-06532R1***

***Men’s and women’s knowledge, attitudes and practices relating to pregnancy and neonatal care: a cross sectional study from Bungoma County, Kenya***

***PLOS ONE***

*Editor’s comments and responses*

The abstract and title still need some editing to reflect the actual research questions and findings. I suggest you emphasise knowledge of danger signs and care seeking practices, rather than the very general 'knowledge, attitudes, and practices.' The abstract completely leaves out care seeking for newborns, which seems to me that it would be the most likely variable to be impacted by increased knowledge of newborn danger signs. Currently, the focus is on ANC utilisation, which as I have noted before, is more likely to be a driver than an impact of increased knowledge.

*Thank you! The title and abstract have been revised to emphasize the study’s focus on neonatal and postnatal care-seeking, and remove the implication that pregnancy care utilization is an outcome.*

Please add to the limitations the potential bias of only speaking to married man (and not women's unmarried partners). The difference in recruitment methods between men and women is explained well; however, it is still not clear why they completed different questionnaires and what the potential implications are.

*Thank you. The Limitations section has been revised to also include the bias of only speaking to married men, and to note the study logistics that determined the use of different questionnaires; also noting that this difference constrains comparability between men’s and women’s answers.*

*Reviewers comments in body of PDF and responses (line numbers refer to PDF)*

Line 111: Reviewer comment: “what about attitudes?”

*Response: The title has been altered to no longer refer to attitudes.*

Line 118: Reviewer comment: “This section should be written to have relevance to the study subject matter. For example, demonstrate how agricultural activities are related to men and women's KAP related to pregnancy and neonatal care. How is the population of the county related to the studied population when this is more of a hospital-based study?”

*Response: The text has been revised to note the ecological relevance.*

Line 127: Reviewer: how were these sample sizes determined/ calculated?

*Response: the convenience sampling has been emphasized in this sentence and below.*

Line 128: Reviewer: state clearly the one year period- between which actual months that constitute the year. This must be known to inform how screening/recruitment was done.

*Response: the period has been specified more accurately*

Line 129: Reviewer: What is the justification for use of this method? this is the poorest method of sampling. it is also inappropriate for sample size and statistical tools are intended for use to determine associations and aimed at generalization.

*Response: We have added text to note the sampling was determined by the resources available to the project team. With this disclosure to inform readers, we believe the subsequent analysis and interpretation is proportionate and holds value.*

Line 130: Reviewer: Reviewer: specify the market centres where recruitment was done- how many and which ones? what is their vicinity in relation to the hospitals?

*Response: Their relationship to the hospitals in the broader study has been specified.*

Line 130: Reviewer: be consistent in use of terms- 'women' instead of 'mothers', in line with your study title.

*Response: we have changed ‘mother’ to ‘women’ or equivalent, throughout, except where needed to specify the parental role.*

Line 130: Reviewer: is it possible that another reason for the selection of these hospitals is because the intervention- the collaborative newborn support project- had taken place at these facilities?

*Response: we have added this detail.*

Line 338: Reviewer: the sampling procedure used- convenience this is not only the weakest type of procedure, but it is also inappropriate for a study sample that is calculated using methods aimed at representativeness.

*Response: we have strengthened our acknowledgement of this limitation*

Line 353: Reviewer: The findings can only be generalized to the target population attending ANC, stretching it to the County when it is a hospital-based study, and using convenience sampling method is misleading.

*Response: We have reworded to avoid the over-generalisation*
